# Surgical Site Infections After Wound Closure With Staples Versus Sutures in Post-renal Transplant Recipients: A Prospective Observational Study

**DOI:** 10.7759/cureus.92197

**Published:** 2025-09-13

**Authors:** Ikram Ullah, Fazle Manan, Muhammad Moosa, Khalil Ur Rehman

**Affiliations:** 1 Department of Urology, Institute of Kidney Diseases, Hayatabad Medical Complex, Peshawar, PAK; 2 Department of Urology and Transplantation, Institute of Kidney Diseases, Hayatabad Medical Complex, Peshawar, PAK

**Keywords:** immunosuppression, renal transplantation, skin closure, staples, surgical site infection, sutures, wound healing

## Abstract

Background

Surgical site infections (SSIs) are a significant postoperative complication, particularly in renal transplant recipients, who are inherently immunosuppressed and prone to delayed wound healing. The choice of skin closure technique may influence infection risk, yet evidence comparing staples versus sutures in this specific population remains limited.

Objective

The main objective of this study is to compare the incidence of SSI after wound closure with staples versus sutures in post-renal transplant recipients.

Methods

This prospective observational study was carried out from January 1, 2022, to January 1, 2024, at the Institute of Kidney Diseases (IKD), Peshawar, Pakistan. Using the skin closure approach, 50 renal transplant recipients were recruited and split into two equal groups of 25 each. We gathered information on immunosuppressive treatment, postoperative problems, comorbidities, demographics, and surgical length. IBM SPSS Statistics for Windows, Version 26 (Released 2019; IBM Corp., Armonk, NY, USA), was used to record and assess the incidence of SSIs during the 30-day postoperative period.

Results

Although the difference was not statistically significant (p = 0.247), the incidence of SSI was higher in the staples group (six patients, 24%) than in the sutures group (two patients, 8%). Additionally, the staples group had higher rates of redness, drainage, and positive wound cultures. Staple closure was linked to a higher - albeit non-significant - adjusted odds ratio for SSI (AOR 3.5; 95% CI: 0.9-12.9; p = 0.07), according to multivariate logistic regression.

Conclusion

While not statistically significant, suture closure demonstrated a trend toward fewer SSIs in renal transplant patients. Sutures may be the preferred method in this high-risk population. Larger trials are warranted.

## Introduction

Renal transplantation remains the definitive treatment for patients with end-stage renal disease (ESRD), significantly improving both survival and quality of life [1]. As surgical techniques and immunosuppressive protocols have advanced over the years, the focus of post-transplant management has increasingly shifted toward minimizing postoperative complications, particularly surgical site infections (SSIs) [2]. SSIs are a critical concern in renal transplant recipients due to their immunosuppressed status, prolonged hospitalization, and multiple comorbidities - all of which predispose them to higher infection rates compared to the general surgical population [3].

SSIs not only compromise graft function, but are also associated with increased morbidity, prolonged hospital stays, and higher healthcare costs [4]. In transplant recipients, even superficial infections can lead to serious outcomes due to impaired wound healing and systemic immunosuppression. Therefore, optimizing every aspect of perioperative care, including wound closure techniques, plays a pivotal role in reducing infection risks [2,5].

Among the various factors contributing to SSIs, the method of skin closure has generated considerable debate. Staples and sutures are the most commonly employed techniques [6]. Staples offer the advantage of quicker application, reduced operative time, and ease of removal [7]. On the other hand, sutures are believed to provide better wound edge approximation and may be associated with lower infection rates in certain patient populations. Evidence from orthopedic and obstetric surgeries has shown mixed results when comparing these methods, with some studies indicating higher SSI rates with staples, while others show no significant difference [8]. However, data regarding their efficacy and safety in renal transplant recipients remain scarce.

Given the unique physiological and pharmacological profiles of post-transplant patients, findings from other surgical disciplines cannot be directly extrapolated to this population. Moreover, inconsistencies in clinical guidelines further underline the need for transplant-specific evidence. The choice of wound closure method often depends on surgeon preference rather than robust data, making it essential to evaluate these techniques in a population already at high risk for infectious complications.

Despite the critical implications of SSIs in renal transplant recipients, there is a notable lack of high-quality, comparative studies assessing the impact of skin closure techniques on postoperative infection rates in this vulnerable group. An evidence-based approach to selecting the most appropriate wound closure method could significantly improve patient outcomes and reduce healthcare burdens. This study is thus designed to bridge this gap by directly comparing the incidence of SSIs following skin closure with staples versus sutures in renal transplant patients, aiming to provide clinicians with concrete data to guide surgical decision-making in transplant settings. The current study aimed to compare the incidence of SSIs following wound closure using staples versus sutures in patients undergoing renal transplantation.

## Materials and methods

This prospective, comparative, observational study was conducted at the Institute of Kidney Diseases (IKD), Peshawar, Pakistan, over two years - from January 1, 2022, to January 1, 2024. Ethical approval was obtained from the MTI-Hayatabad Medical Complex Ethical Review Board (approval number: 1731). Before inclusion in the study, each subject provided informed written consent.

The sample size was calculated using OpenEpi version 3.01, considering a confidence level of 95%, a power of 80%, and an expected difference in infection rates between the two groups: 11.7% for sutures and 30.0% for staples [9]. Based on these assumptions, a total of 50 participants were required, with 25 allocated to each group.

A non-probability consecutive sampling method was used to choose the patients. Adult patients between the ages of 18 and 65 undergoing elective kidney transplantation who had their skin closed with non-absorbable sutures or staples met the inclusion criteria. Exclusion criteria included patients undergoing multi-organ transplantation, those with pre-existing wound infections, re-transplantation cases, patients already on systemic antibiotics for other infections, or those with uncontrolled diabetes (HbA1c > 8%), or coagulopathies.

Patients were split into two groups according to the wound closure technique the surgeon chose at the time of the procedure. In Group A, surgical wounds were closed using stainless-steel staples, while in Group B, wounds were closed using non-absorbable monofilament sutures (Prolene® or Nylon® 2-0) with an interrupted technique. All transplant surgeries were performed under standard aseptic conditions by experienced transplant surgeons. Preoperative antibiotic prophylaxis with intravenous cefazolin (1 g) was administered 30 to 60 minutes before skin incision in all patients, following institutional protocol.

Postoperatively, patients were monitored daily during their mean hospital stay of 7.2 ± 1.3 days, and subsequently followed up at outpatient visits on days 7, 14, and 30. SSIs were identified using CDC criteria [10], which included purulent wound discharge, localized signs of inflammation such as erythema and swelling, positive wound cultures, or clinical diagnosis by the treating physician. A structured proforma was used to record patient demographics, comorbidities (such as diabetes and hypertension), hospital stay duration, type of immunosuppressive therapy, surgical duration, and any signs of infection or complications (Table 6, see Appendix). Wound swabs for culture and sensitivity were sent in suspected cases of SSI, and infections were managed as per hospital protocol.

The analysis of the data was done with IBM SPSS Statistics for Windows, Version 26 (Released 2019; IBM Corp., Armonk, NY, USA). While categorical data, like gender and the incidence of SSI, were given as frequencies and percentages, continuous variables, like age and body mass index (BMI), were shown as mean ± standard deviation. The incidence of SSIs between the two groups was compared using the Chi-square test, also known as Fisher's exact test, while continuous variables were compared using the independent samples t-test. A p-value below 0.05 was regarded as statistically significant. Multivariate logistic regression analysis was used to find independent predictors of SSI, while controlling for potential confounding variables such as diabetes status, BMI, surgical duration, and type of immunosuppressive therapy.

## Results

The study involved 50 patients in total, 25 of whom were in the staples group, and 25 of whom were in the sutures group. Participants in the staples group were 42.1 ± 9.8 years old on average, whereas those in the sutures group were 41.3 ± 8.6 years old on average. Age differences between the two groups were not statistically significant (p = 0.548). In terms of gender distribution, there were 17 males (68%) and 8 females (32%) in the staples group, while there were 18 males (72%) and 7 females (28%) in the sutures group. There was no statistically significant variation in the gender distribution across the groups (p = 0.629). The mean BMI was slightly higher in the staples group (27.3 ± 2.9 kg/m²) compared to the sutures group (26.9 ± 3.1 kg/m²); however, this difference did not reach statistical significance (p = 0.089) (Table 1).

**Table 1:** Demographic and Clinical Characteristics of Study Participants (n = 50) Values are presented as mean ± SD or n (%). p-values were calculated using the independent samples t-test for continuous variables, and the Chi-square test for categorical variables. BMI, Body Mass Index

Variable		Staples Group (n = 25)	Sutures Group (n = 25)	Test Statistic	p-value
Age (years)	Mean ± SD	42.1 ± 9.8	41.3 ± 8.6	t = 0.61	0.548
Gender; n (%)	Male	18 (72%)	17 (68%)	χ² = 0.23	0.629
	Female	7 (28%)	8 (32%)		
BMI (kg/m²)	Mean ± SD	27.3 ± 2.9	26.9 ± 3.1	t = 1.72	0.089

The two groups had comparable rates of common comorbidities. Ten patients (40%) in the staples group and nine patients (36%) in the sutures group had diabetes mellitus; nevertheless, there was no statistically significant difference between the two groups (p = 0.099). A similar comorbidity burden was seen in both groups, with 13 patients (52%) in the sutures group and 14 patients (56%) in the staples group reporting hypertension (p = 0.987). Regarding immunosuppressive treatment, tacrolimus, mycophenolate mofetil (MMF), and prednisolone were administered to most patients in both groups: 20 patients (80%) in the staples group and 21 patients (84%) in the sutures group (p = 0.999). Five patients (20%) in the staples group and four patients (16%) in the sutures group had lower rates of cyclosporine, MMF, and prednisolone use (p = 0.989). Additionally, the average surgery time was similar for both groups, averaging 145.2 ± 15.6 minutes for the staples group and 143.8 ± 14.7 minutes for the sutures group (p = 0.988) (Table 2).

**Table 2:** Comorbidities, Immunosuppressive Therapy, and Surgical Duration of Study Participants (n = 50) p-values were calculated using the Chi-square test for categorical variables, and the independent samples t-test for continuous variables. MMF, Mycophenolate Mofetil

Variable	Staples Group (n = 25)	Sutures Group (n = 25)	Test Statistic	p-value
Diabetes Mellitus	10 (40%)	9 (36%)	χ² = 2.72	0.099
Hypertension	14 (56%)	13 (52%)	χ² = 0.01	0.987
Tacrolimus + MMF + Prednisolone	20 (80%)	21 (84%)	χ² = 0.00	0.999
Cyclosporine + MMF + Prednisolone	5 (20%)	4 (16%)	χ² = 0.00	0.989
Mean Surgical Duration (minutes)	145.2 ± 15.6	143.8 ± 14.7	t = 0.11	0.988

The staples group experienced higher postoperative problems than the sutures group, although the differences were not statistically significant. Five patients (20%) in the staples group and two patients (8%) in the sutures group experienced redness and indications of local inflammation at the surgical site (p = 0.415). Three patients (12%) with staple closure developed purulent wound discharge, while only one patient (4%) in the suture group showed this symptom (p = 0.602). Furthermore, two patients (8%) in the staples group and one patient (4%) in the sutures group had positive wound cultures, confirming infection (p = 0.988) (Table 3).

**Table 3:** Postoperative Signs of Infection and Complications of Study Participants (n = 50) p-values were calculated using the Chi-square test.

Variable	Staples Group (n = 25)	Sutures Group (n = 25)	Test Statistic	p-value
Redness/Inflammation	5 (20%)	2 (8%)	χ² = 0.66	0.415
Purulent Discharge	3 (12%)	1 (4%)	χ² = 0.27	0.602
Positive Wound Culture	2 (8%)	1 (4%)	χ² = 0.00	0.988

An overall incidence of 16% was obtained from the observation of SSIs in 8 out of 50 patients. Six patients (24%) in the staples group experienced SSIs, while only two patients (8%) in the sutures group did so. Although the staples group seemed to have a higher incidence, the difference was not statistically significant (p = 0.247). With 19 patients (76%) in the staples group and 23 patients (92%) in the sutures group exhibiting no symptoms of postoperative infection, the majority of patients in both groups remained infection-free (Table 4).

**Table 4:** Incidence of SSIs in Study Participants (n = 50) p-values were calculated using the Chi-square test. SSI, Surgical Site Infection

Group	SSI Present	SSI Absent	Test Statistic	p-value
Staples	6 (24%)	19 (76%)	χ² = 1.34	0.247
Sutures	2 (8%)	23 (92%)		
Total	8 (16%)	42 (84%)		

To find independent predictors of SSI, multivariate logistic regression analysis was used. Some of the variables showed a tendency toward higher risk, but none of them achieved statistical significance (p > 0.05). With an adjusted odds ratio (AOR) of 3.5 (95% CI: 0.9-12.9; p = 0.07), staple closure was linked to a greater risk of SSI than suture, indicating a potential relationship deserving of more research. Although they did not reach statistical significance, other variables that also showed greater chances for SSI were diabetes mellitus (AOR: 2.1; 95% CI: 0.5-8.4; p = 0.31), obesity (BMI ≥ 30; AOR: 1.8; 95% CI: 0.4-7.5; p = 0.45), and prolonged surgical length (>150 minutes; AOR: 2.6; 95% CI: 0.6-11.2; p = 0.21) (Table 5).

**Table 5:** Multivariate Logistic Regression Analysis of Risk Factors for SSI in Study Participants (n = 50) SSI, Surgical Site Infection; BMI, Body Mass Index

Variable	Adjusted Odds Ratio (AOR)	95% CI	p-value
Staple Closure (vs. Suture)	3.5	0.9-12.9	0.07
Diabetes Mellitus	2.1	0.5-8.4	0.31
BMI ≥ 30	1.8	0.4-7.5	0.45
Surgical Duration > 150 minutes	2.6	0.6-11.2	0.21

**Figure 1:**
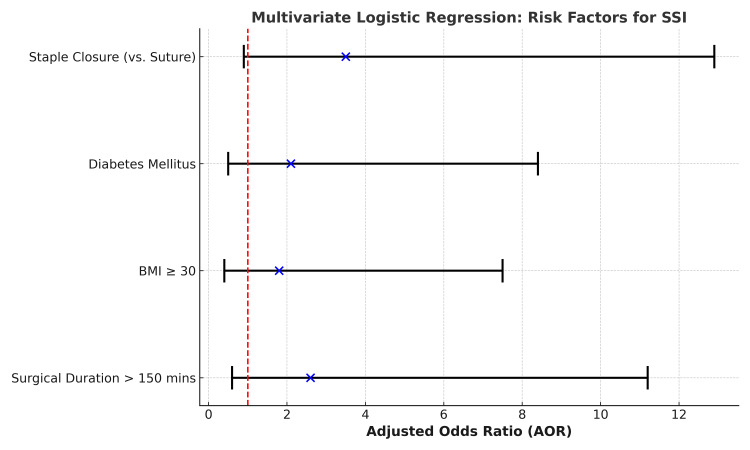
Graph Showing the AORs With 95% Confidence Intervals for the Identified Risk Factors of SSI The red dashed line at AOR = 1 indicates the threshold of no effect. SSI, Surgical Site Infection; AORs, Adjusted Odds Ratios; BMI, Body Mass Index

Figure 1 illustrates the AORs with 95% CIs for potential risk factors of SSI. Staple closure showed the highest estimated risk (AOR: 3.5), followed by prolonged surgical duration (>150 minutes), diabetes mellitus, and BMI ≥ 30; however, none of these associations reached statistical significance, as all confidence intervals crossed the line of no effect (AOR: 1).

## Discussion

In this prospective comparative study of 50 renal transplant recipients, we found that the incidence of SSI was higher in the group with staple closure (24%) compared to the suture group (8%). Although this difference was not statistically significant (p = 0.247), the trend was consistent across multiple postoperative parameters. Signs of local inflammation, purulent discharge, and positive wound cultures were observed more frequently in patients whose wounds were closed with staples. Multivariate analysis further supported this trend, showing an AOR of 3.5 for SSI associated with staple use, though the result narrowly missed statistical significance (p = 0.07). Demographic and clinical characteristics - including age, gender, BMI, comorbidities, immunosuppressive regimens, and surgical duration - were well balanced between the two groups, indicating that the observed differences in infection rates were likely attributable to the wound closure method.

These findings resonate with a 2022 meta-analysis of elective hip and knee arthroplasty, which found that staple closure carried a higher relative risk for SSI compared to suturing (RR: ~1.70), particularly significant in hip arthroplasty (RR: 2.51, 95% CI: 1.15-5.50) [11,12]. Similarly, a broader network meta-analysis encompassing gastrointestinal surgeries concluded that absorbable sutures were associated with significantly lower SSI rates compared to skin staplers (OR: 0.77, 95% CI: 0.63-0.95) [13].

In the field of orthopedics, continuous intracutaneous sutures versus staples in spine surgery showed comparably low SSI rates (~1 per group), yet staples offered faster closure times [14,15]. Likewise, in elective abdominal surgeries performed in Pakistan in 2024, although staples shortened closure time significantly (p = 0.004), SSI rates did not differ significantly between methods; prolonged wound discharge and pain were, however, notably higher in the staples group - consistent with our observation of increased local inflammation and discharge in stapled wounds, though not statistically significant [14].

A randomized trial in diabetic patients undergoing knee arthroplasty in 2025 found no significant difference in wound infection or dehiscence between sutures and staples (p ≈ 0.254), aligning with our non-significant findings; however, factors like BMI and HbA1c were significant predictors of dehiscence and infection in that cohort [16]. This supports our multivariate results, where high BMI and diabetes status, although nonsignificant, were associated with elevated odds of SSI (AOR: 1.8 and 2.1, respectively).

In the Pakistani setting, a randomized trial comparing staples versus sutures in inguinal hernioplasty in 2024 reported a significantly lower SSI rate with staples (3%) than sutures (12%, p = 0.012) [17]. These divergent results may reflect differences in operative site, tissue tension, microbial flora, or patient population, underlining the importance of procedure- and context-specific evidence. From 2025 consensus reviews in orthopedics, it is noted that staples are associated with faster closure times but higher pain, and inferior perfusion and aesthetic outcomes than continuous suture techniques (improved dermal apposition and microvascular flow with sutures) [15]. These procedural differences may partially explain the trend toward higher SSI in stapled closures, even in transplant recipients.

In contrast, the 2021 meta-analysis in open abdominal surgery in the digestive system found no statistically significant difference between subcuticular sutures and staples for SSI prevention (OR: 0.81, p = 0.15) [18]. This aligns with our finding of no significant difference, although our cohort showed a numeric advantage with sutures. Taken together, these studies suggest that suture techniques may offer modest advantages in infection control, particularly in scenarios with higher tension or compromised healing (e.g., transplantation), while some clean, low-risk surgeries may not show differences in SSI. Overall, our results fall broadly in line with recent literature: while staples may reduce operative time, they tend to carry a higher, though sometimes statistically nonsignificant, risk of superficial infection compared to sutures. Given the immunocompromised status of transplant recipients, these differences may clinically matter more than they would in low-risk populations.

According to the study's findings, suture closure, as opposed to staple closure, may be linked to a decreased risk of SSIs in patients of kidney transplants. The observed trend is clinically relevant, even though the difference was not statistically significant - especially in this immunocompromised population, where even small infections might result in major consequences. The choice of wound closure method, often based on convenience or surgeon preference, should instead be guided by patient risk factors and emerging clinical evidence. Given that renal transplant patients are particularly vulnerable to postoperative infections due to immunosuppressive therapy, using a closure technique that minimizes infection risk, such as interrupted non-absorbable sutures, could potentially improve wound healing outcomes and reduce hospital readmissions and long-term morbidity.

There are various limitations to this study. First, the capacity to identify statistically significant differences may have been limited by the sample size, which was rather small. Second, because just one tertiary care facility was used for the study, the results might not apply to other groups or establishments that use various surgical techniques. Third, the surgeon chose the closure technique for each patient, rather than assigning them at random, which could lead to selection bias. Additionally, factors such as postoperative wound care, patient adherence to instructions, and variations in individual immune responses could not be fully standardized or controlled.

## Conclusions

In conclusion, while the difference in SSI rates between staple and suture closure in renal transplant recipients was not statistically significant, the trend observed favors sutures as the safer option with fewer infectious complications. These findings underscore the importance of surgical decision-making in high-risk populations and highlight the need for larger, multicenter, randomized controlled trials to establish definitive recommendations. Until such data are available, clinicians should weigh the risks and benefits of each closure method carefully, especially when managing immunosuppressed patients.

## Appendices

Data collection tool

**Table 6:** Data collection tool

Section	Item	Details/Response Options
A. Demographics	Name/Initials	____________________
	Age (years)	_________
	Gender	☐ Male ☐ Female
	Height (m)	________
	Weight (kg)	________
	BMI	_________
B. Clinical Profile	Diabetes Mellitus	☐ Yes ☐ No
	HbA1c (if diabetic)	_________
	Hypertension	☐ Yes ☐ No
	Other Comorbidities	________________________
C. Transplant & Surgical Details	Type of Closure	☐ Group A – Staples ☐ Group B – Sutures
	Duration of Surgery (minutes)	_________
	Duration Category	☐ ≤150 ☐ >150
	Surgeon Initials	___________
	Pre-op Antibiotic	☐ Yes ☐ No
D. Immunosuppressive Regimen	Regimen	☐ Tacrolimus + MMF + Prednisolone ☐ Cyclosporine + MMF + Prednisolone ☐ Other: ____________________
E. Postoperative Monitoring & Complications	Date	Redness/Inflammation
	_______	☐ Yes ☐ No
	_______	☐ Yes ☐ No
	_______	☐ Yes ☐ No
F. Surgical Site Infection – CDC Criteria	SSI Criteria (tick all that apply)	☐ Purulent discharge ☐ Local erythema/swelling/inflammation ☐ Positive wound culture ☐ Clinical diagnosis by surgeon
	Final SSI Diagnosis	☐ Present ☐ Absent
	Date of SSI Diagnosis	___________
G. SSI Management	Wound Swab Sent for C/S	☐ Yes ☐ No Date: _________
	Antibiotics Given	☐ Yes ☐ No Name: ____________________
	Other Management	__________________________________
H. Predictors for SSI (Risk Factor Coding)	Closure Type	☐ Staples ☐ Sutures
	Diabetes Mellitus	☐ Yes ☐ No
	BMI ≥ 30	☐ Yes ☐ No Value: _________
	Surgical Duration >150 min	☐ Yes ☐ No
